# Paternal health in the first 12-13 years of the ALSPAC study

**DOI:** 10.12688/wellcomeopenres.18639.2

**Published:** 2024-03-21

**Authors:** Jean Golding, Iain Bickerstaffe, Yasmin Iles-Caven, Kate Northstone

**Affiliations:** 1 Centre for Academic Child Health, Bristol Medical School, University of Bristol, Bristol, BS8 2BN, UK; 2 Population Health Sciences, Bristol Medical School, University of Bristol, Bristol, BS8 2BN, UK

**Keywords:** ALSPAC, paternal health, father, morbidity, pain, anxiety, depression, fatigue

## Abstract

The Avon Longitudinal Study of Parents and Children (ALSPAC) collected information from the enrolled pregnancy onwards to identify features of the environment in which the study child was brought up. Among data collected were features concerning the health of the mothers’ partners – generally the study father. This was an important feature since the father’s physical and mental health can have a long-term effect on the family. In this Data Note we describe the data available on the father’s health from pregnancy until 12 years after the offspring was born. Not only is this a valuable addition to the environmental information available for studies of the child’s development and the mental health of the mother over time, but it will provide a useful description of the father himself during adulthood.

## Introduction

The Avon Longitudinal Study of Parents and Children (ALSPAC) was designed to assess ways in which the health, well-being and development of the child was affected by the environment in which they grew up ( Boyd *et al.*, 2013; Fraser *et al.*, 2013; Golding *et al.*, 2001). One of the environmental features on which data were collected included the physical and mental health of the mother’s partner. These data, which were collected at various stages during pregnancy and the child’s early life, provide information which will be valuable in regard to the study’s initial aims, but also provides a useful longitudinal resource concerning the health and wellbeing of fathers over time. Thus, in this Data Note we describe the physical and mental health of the mother’s partner for the period from the middle of pregnancy until 12–13 years after the birth of the study child. All the data were collected by questionnaire – from both **the mother’s partner (for simplicity hereafter referred to as the study father)**, and from the study mother - in regard to the health of her partner. The data are available to interested researchers. 

## Material and methods

### The ALSPAC sample

Pregnant women residents in Avon, UK with expected dates of delivery 1st April 1991 to 31st December 1992 were invited to take part in the study. The initial number of pregnancies enrolled was 14,541 (for these at least one questionnaire has been returned or a “Children in Focus” clinic had been attended by 19/07/99). Of these initial pregnancies, there was a total of 14,676 fetuses, resulting in 14,062 live births and 13,988 children who were alive at 1 year of age.

*Details of study father* (mother’s partner)

There are several features to note:

(i) The mother was asked at each stage from pregnancy until the child was aged 12 years, to hand a questionnaire to her partner if she was happy for him to take part. This included its own reply-paid envelope so that this could be returned by the partner in confidence.
(ii) The mother was asked to define her partner – and was told that it was not essential that this be the biological father of the child. However, **for simplicity her partners are referred to throughout this paper as the study fathers, regardless of their gender or parental status**.
(iii) The fact that some of the study fathers are not the biological father is important to note – but it was intentional that the study took account of the characteristics of the person the mother looked to as her partner and who, more often than not, took the place of the child’s father. Partners may change over time, but information on this is documented elsewhere (see details of the PZ file in the ALSPAC Data Dictionary).
(iv) It should be noted that the data were collected relative to the age of the study child; thus, where there is more than one study child, the father will have two entries. This may be important to keep separate when considering the associations with child outcomes, as well as with those of the father. The 52 instances where the fathers are related to two pregnancies can be distinguished using the variable pz_mult. It should be noted also that, for multiple births such as twins, the parents (mothers and fathers) each received only one questionnaire per time point. This is in contrast to data collected on the children themselves for which there were individual questionnaires related to each child.
(v) Information is available at most individual timepoints to distinguish the fathers who lived with the mother, those who were married to her, those who were not male and those who considered that they were the study child’s biological father.

### The presentation of the information on data available

The documentation below concerns the data that were collected and for which the frequencies of responses were published online in 2021 in the released data files published on the ALSPAC website Researchers | Avon Longitudinal Study of Parents and Children | University of Bristol. The actual numbers available may be slightly lower than shown since, over time, a very small number of participants withdrew consent for their data to be used. The tables in this Data Note include the variable name, the time after delivery that the questionnaire was administered, the numbers of participants responding to the specific question, and the % responding to each category after any missing responses have been excluded. Where available, responses from the study mother concerning the father’s health are also included.

We recommend that researchers wishing to use these data always refer to the actual question used as these sometimes changed slightly between questionnaires. The best way to do this is to refer to the questionnaires as published in the ALSPAC Data Dictionary and view the copy of the actual questions asked as well as the ways in which they have been coded as described in the relevant documentation. http://www.bristol.ac.uk/alspac/external/documents/ALSPAC_Data_Dictionary.zip

The data collected are described hereafter as follows: (1) general measures of health; (2) specific aspects of physical health; (3) psychiatric and neurological conditions; and (4) other health data available. For the non-clinician, we have described the condition(s) briefly under each section.

**1. General measures of health**

*1.1 Subjective measures of health*

Subjective measures of health are frequently collected in questionnaire-based studies and self-reported ill health has often been shown to be a good predictor of long-term adverse consequences (including mortality) ( Sajjad *et al.*, 2017). In ALSPAC, information on how healthy the study father was feeling was collected at various times from both the mother (8 time points from pregnancy to 12 years after the child was born) and the father (8 time points from 8 months after the child was born). In general, most of the fathers were reported as being fit and well, but it is notable that mothers increasingly reported them to be ‘often unwell’ or ‘hardly ever well’ over time. This was not apparent among the fathers’ own reports which was likely due to those who were unwell not being given the questionnaire to complete ( Table 1.1).

**Table 1.1.** General measures of health of study fathers as provided by the father and by the mother herself.

Time asked	Variable name	N	Always F & W %	Mostly F & W %	Often unwell %	Hardly ever well %
*Maternal report*						
Preg	A524	13026	46.8	48.9	3.8	0.4
8m	F510	10640	50.4	46.2	3.0	0.4
21m	G600	9704	48.5	48.3	3.0	0.2
33m	H485	8951	43.1	52.3	4.3	0.3
4y	J603	8740	46.6	48.9	4.0	0.4
6y	L6010	7894	39.8	54.0	5.6	0.7
9y	P3010	7300	42.2	51.4	5.8	0.6
12y	S3010	6355	32.5	60.0	6.6	0.8
*Paternal report*						
8m	PD010	6843	44.4	52.6	2.5	0.5
21m	PE010	6143	61.9	35.5	2.4	0.2
33m	PF1000	5317	62.8	34.2	2.7	0.3
4y	PG1000	5078	62.2	34.8	2.7	0.3
5y	PH1000	4484	62.4	34.4	2.8	0.4
6y	PJ3000	4438	56.0	39.8	3.8	0.3
9y	PM1000	3656	65.0	32.1	2.5	0.3
12y	PQ1000	3267	62.6	34.1	2.8	0.6

Preg = pregnancy; F & W = fit and well; N = no. completed.

*1.2. Reports of (non-specific) illness*

The life events inventory administered at 9 intervals to both the mother and father included a question on whether the father had been ill (or very ill) during the period of time considered, and if so, how much it had affected the respondent. Table 1.2 shows that the mother reported that her partner had been ill for between 11 and 20% of the time periods. However, the father himself reported illness far less frequently (3 – 6%), perhaps not surprising as the wording of *his* life event scale concerned whether he was ‘very’ ill. There was little evidence of increasing or decreasing rates of illness over time.

**Table 1.2.** Illness (not specified) of father reported within life events inventory (N.B. father’s statement was that he was ‘very ill’; the mothers just referred to him being ill).

Time asked	variable name	N	Period covered	Yes %	No %
*Maternal report*					
Mid-pregnancy	B574	11982	1 ^st^ half of pregnancy	16.4	83.6
8 months	F224a	11194	2 ^nd^ half preg to 8 months	16.3	83.7
21 months	G304a	10235	Since 8 months	20.3	79.7
33 months	H214a	9635	Since 18 months	19.8	80.2
4 years	J304a	9501	Since 30 months	18.5	81.5
5 years	K4004	8894	In past year	19.2	80.8
6 years	L4004	8469	Since 5 ^th^ birthday	15.1	84.9
9 years	P2004	7818	Since 6 ^th^ birthday	12.9	87.1
11 years	R5004	7508	Since 9 ^th^ birthday	11.3	88.7
*Paternal report*					
Mid-pregnancy	PB170a	10000	1 ^st^ half of pregnancy	3.2	96.8
8 months	PD230a	6861	2 ^nd^ half preg to 8 months	4.0	96.0
21 months	PE310a	6156	Since 8 months	5.7	94.3
33 months	PF5010	5385	Since 18 months	5.6	94.4
4 years	PG3010	5077	Since 30 months	5.4	94.6
5 years	PH4010	4480	In past year	5.8	94.2
6 years	PJ4010	4438	Since 5 ^th^ birthday	5.1	94.9
9 years	PM2010	3610	Since 6 ^th^ birthday	4.4	95.6
11 years	PP5010	3597	Since 9 ^th^ birthday	4.1	95.9

N = no. completed

*1.3. Hospital admissions*

Hospital admission data was also collected as part of the father’s life event scales, but her partners’ admissions were not included in the mother’s scales, The available information is described in Table 1.3. The data are non-specific and do not have any accompanying details such as whether or not any admission was an emergency, the reason for admission or length of stay.

**Table 1.3.** Hospital admission (reason not specified) of father reported within life events inventory.

Time asked	Variable name	N	Period covered	Yes %	No %
*Paternal report*					
Mid-pregnancy	PB166a	10000	1 ^st^ half of pregnancy	2.8	97.2
8 months	PD226a	6861	2 ^nd^ half preg to 8 months	3.2	96.8
21 months	PE306a	6156	Since 8 months	4.9	95.1
33 months	PF5006	5381	Since 18 months	6.2	93.8
4 years	PG3006	5077	Since 30 months	6.9	93.1
5 years	PH4006	4485	In past year	6.3	93.7
6 years	PJ4006	4435	Since 5 ^th^ birthday	7.0	93.0
9 years	PM2006	3609	Since 6 ^th^ birthday	12.7	87.3
11 years	PP5006	3597	Since 9 ^th^ birthday	9.6	90.4

N = no. completed

*1.4. Use of regular medication*

One further non-specific measure of general ill-health concerns whether the father was taking medication regularly for health reasons. This was asked on 3 occasions and shows a sharp increase from 13.6% responding affirmatively during the index pregnancy to 23.5% 11 years later ( Table 1.4).

**Table 1.4.** The father was currently taking medication regularly for health reasons.

Time asked	Variable name	N	Yes %	No %
Pregnancy	PA200	8433	13.6	86.4
8 years	PL1090	3852	19.7	80.3
11 years	PP2050	3531	23.5	76.5

N = no. completed

**2. Physical health**

***2.1. Conditions resulting in joint and skeletal pain***

*2.1a. Arthritis*

Arthritis is the swelling and tenderness of one or more joints. The main symptoms are joint pain and stiffness, which typically worsen with age. The most common type of arthritis is osteoarthritis. The prevalence of a history of arthritis in the fathers increased over time from 4.5% during the index pregnancy to 9.2% eleven years later ( Table 2.1aa). It can be seen that a similar rise in prevalence of consultations for the disorder occurred gradually over time, with similar rates being reported by mothers and their partners ( Table 2.1ab).

**Table 2.1aa.** History of whether father had ever had arthritis distinguishing between recently (in past year) and prior to the past year only.

Time asked	Variable name	N	Yes		No
			Recently %	In past %	Never %
Pregnancy	PA183	8382	2.9	1.6	95.5
8 years	PL1053	3964	5.8	2.3	91.9
11 years	PP2013	36109	6.3	2.9	90.8

**Table 2.1ab.** Prevalence (%) of arthritis in study fathers and whether they consulted a doctor during particular periods since the birth of the study child.

Time asked	Variable Name	N	Period covered	Yes consulted Dr		Not at all
				Yes %	No %	%
*Paternal report*						
8m	PD033	6830	Since baby was born	0.7	1.1	98.2
21m	PE033	6151	Since baby was 8m	1.1	1.1	97.8
4y	PG1023	4920	In past year	1.5	1.7	96.8
5y	PH1028	4423	In past year	1.7	1.7	96.6
6y	PJ3028	4430	Since child’s 5 ^th^ birthday	1.9	2.3	95.8
9y	PM1028	3613	Since child’s 6 ^th^ birthday	2.5	3.1	94.4
12y	PQ1028	3201	In the last 2 years	2.8	3.1	94.1
*Maternal record*						
8m	F531	10615	Since baby’s birth	0.7	0.7	98.6
21m	G618	8961	Since baby was 8m	1.1	1.1	97.8
33m	H503	9026	Since baby was 18m	1.6	1.0	97.4
4y	J621	8414	In past year	1.5	1.2	97.3
6y	L6037	7467	Since child was 5	1.9	1.7	96.4
9y	P3037	7251	In last 2 years	2.2	1.8	96.0
11y	S3037	6542	Since child’s 10 ^th^ birthday	2.2	2.2	95.6

*2.1b. Rheumatism*

Rheumatism is a term that people often used in the past when describing pain and other symptoms affecting the muscles and joints. Healthcare professionals tend not to use this term, but they use similar ones, such as “rheumatoid”. When people use the term, they often mean Rheumatoid arthritis, which is an inflammatory autoimmune condition that leads to swelling in the joints. Compared with arthritis the pattern for rheumatism showed a less consistent increase over time, changing from 4.1% at the time of pregnancy to 4.5% after a further 11 years ( Table 2.1ba). The breakdown by short time periods appears to show an increase over time, but it should be remembered that comparing the rates in Table 2.1bb is prone to error since the time intervals are often not comparable.

**Table 2.1ba.** History of whether father had ever had rheumatism distinguishing between recently (in past year) and prior to the past year only.

Time asked	Variable Name	N	Yes		No
			Recently %	In past %	Never %
Pregnancy	PA182	3988	2.0	2.2	95.9
8 years	PL1052	3956	2.8	2.1	95.1
11 years	PP2012	3614	2.3	2.2	95.5

N = no. completed

**Table 2.1bb.** Prevalence (%) of rheumatism in study fathers and if so whether they consulted a doctor.

Time asked	Variable name	N	Period covered	Yes Consulted Dr.		Not at all
				Yes >%	No >%	%
*Paternal record*						
8m	PD034	6830	Since baby was born	0.2	1.0	98.7
21m	PE034	6151	Since baby was 8m	0.4	1.0	98.7
4y	PG1024	4910	In past year	0.5	1.5	98.1
5y	PH1029	4419	In past year	0.6	1.6	97.9
6y	PJ3029	4421	Since child’s 5 ^th^ birthday	0.7	1.8	97.5
9y	PM1029	3609	Since child’s 6 ^th^ birthday	0.6	2.0	97.4
12y	PQ1029	3199	In the last 2 years	0.8	1.8	97.4
*Maternal record*						
8m	F534	10615	Since baby’s birth	0.2	0.7	99.1
21m	G620	8931	Since baby was 8m	0.4	0.7	98.9
33m	H505	9026	Since baby was 18m	0.4	0.8	98.9
4y	J623	8397	In past year	0.5	0.8	98.7
6y	L6039	7455	Since child was 5	0.7	1.2	98.1
9y	P3039	7246	In last 2 years	0.8	1.3	97.9
11y	S3039	6526	Since child’s 10 ^th^ birthday	0.7	1.2	98.1

N = no. completed

*2.1c. Back pain*

Back pain was recorded on a number of occasions, but its description often varied slightly – for example with inclusion of terms such as slipped disc. The prevalence of back pain was very commonly reported by fathers, especially when considering claims of pain occurring recently. This varied from 19% to 28% over a period of 12 years ( Table 2.1ca). The paternal reported frequency of back pain showed no consistent variation ( Table 2.1cb), but maternal report did show an apparent increase over time ( Table 2.1cc).

**Table 2.1ca.** History of whether father had ever had back pain distinguishing between recently (in past year) and prior to the past year only.

Time asked	Variable name	N	Yes		No
			Recently %	In past %	Never %
Pregnancy	PA178	8501	18.5	28.3	53.2
8 years	PL1048	3985	28.8	30.4	40.8
11 years	PP2008	3624	27.8	32.0	40.1

N = no. completed

**Table 2.1cb.** Frequency (%) of back ache in study fathers in the past month.

Time asked	Variable Name	N	Always	Sometimes	Not at all
21m *	PE110	6140	3.5	34.4	62.1
4y	PG1130	4995	8.5	42.5	49.0
5y *	PH1150	4289	4.1	37.0	58.9
6y	PJ3240	4308	5.2	39.8	55.2
9y	PM1190	3834	3.7	38.9	57.3
11y	PP2140	3618	5.9	45.4	48.7
12y	PQ1190	3258	5.2	41.3	53.5

N = no. completed; *Question included an option ‘once only’. This has been combined with ‘sometimes’

**Table 2.1cc.** Prevalence (%) of back ache in study fathers and if so whether they consulted a doctor.

Time asked	Variable name	N	Period covered	Yes consulted Dr		Not at all
				Yes %	No %	%
*Paternal record*						
8m	PD023	6826	Since baby born	4.2	32.7	63.1
21m	PE023	6151	Since baby was 8m	6.9	35.5	57.6
4y	PG1013	4995	In past year	8.5	42.5	49.0
5y	PH1014	4410	In past year	8.3	27.8	64.1
6y	PJ3014	4429	Since child was 5y	9.9	28.4	61.7
9y	PM1014	3626	Since child’s 6 ^th^ birthday	13.9	31.2	54.9
12y	PQ1014	3222	In the last 2 years	12.3	33.4	54.4
*Maternal record*						
33m	G621	9066	In past year	9.6	18.7	71.7
4y	H506	9026	In past year	10.9	19.6	69.5
4y	J624	8420	Since child’s 5 ^th^ birthday	10.9	21.9	67.2
6y	L6040	7753	Since child was 5	11.1	25.2	63.7
9y	P3040	6711	In last 2 years	15.0	26.9	58.1
11y	S3040	6696	In the last 2 years	11.2	25.8	63.0

N = no. completed

*2.1d. Other joint aches and pains*

The frequencies with which the fathers reported knee pain, neck ache or shoulder ache are shown in Table 2.1d. The specific aches/pains that were reported as always present increased over time, but at no point exceeded 3% of the population of fathers.

**Table 2.1d.** Frequency (%) of other aches and pains in the past month in study fathers.

Time asked	Variable name	N	Always %	Sometimes %	Not at all %
*Knee pain*					
5y *	PH1171	3554	2.5	18.2	79.5
9y **	PM1220	3662	20.3		79.7
*Neck ache*					
21m *	PE129	6140	1.0	24.0	75.0
4y	PG1149	5027	1.4	26.6	72.0
5y *	PH1169	4475	1.5	26.6	71.9
6y	PJ3259	4435	1.8	26.8	71.5
9y	PM1209	3642	1.4	26.9	71.7
11y	PP2159	3620	2.0	29.0	69.0
12y	PQ1209	3243	2.0	28.4	69.6
*Shoulder ache*					
21m *	PE126	6140	1.1	19.5	79.4
4y	PG1146	5033	1.7	21.6	76.7
5y *	PH1166	4475	1.9	21.4	76.8
6y	PJ3256	4438	2.1	23.3	74.7
9y	PM1206	3638	1.5	23.6	74.9
11y	PP2156	3619	2.9	24.7	72.4
12y	PQ1206	3240	2.6	24.9	72.5

N = no. completed; *Question included an option ‘once only’; This has been combined with ‘sometimes’; ** Question only enquired yes/no.

**2.2. *Respiratory conditions***

*2.2a. Asthma and wheezing*

Asthma is a condition in which airways are narrowed and swollen and may produce extra mucus. This can make breathing difficult and trigger coughing, a whistling sound (wheezing) when breathing out and shortness of breath. For some people, asthma is a minor nuisance; for others, it can be a major problem that interferes with daily activities and may lead to a life-threatening asthma attack.

Fathers were asked on three occasions whether they had ever had asthma, distinguishing between occurrences during the previous year and earlier in time ( Table 2.2aa). There is some evidence that asthma increased over time (6.3% reported in pregnancy compared to 9–10% later).

**Table 2.2aa.** History of whether father had ever had asthma distinguishing between those who had had asthma in the past year and those who had had asthma but not in the past year.

Time asked	Variable Name	N	Yes		No
			Recently %	In past %	Never %
Pregnancy	PA173	8478	6.3	6.5	87.2
8 years	PL1043	3970	9.8	7.7	82.4
11 years	PP2003	3624	8.6	7.5	83.9

N = no. completed

The frequency with which fathers reported wheezing with whistling on the chest was collected at the same three time points, and such a history varied from 18.4% to 16.8% over time, but with no indication of any increase ( Table 2.2ab).

**Table 2.2ab.** Frequency of wheezing with whistling on the chest in past 2 years by study fathers.

Time asked	Variable name	N	Often %	Sometimes %	No %
Pregnancy	PA220	8493	3.7	14.7	81.6
8 years	PL1110	3960	3.2	15.3	81.4
11 years	PP2070	3600	2.0	14.8	83.2

N = no. completed

The occurrence of asthma like symptoms in the father was also asked of both parents covering 7 specific periods of time. This involved asking about ‘asthma or wheezing’ – and whether this had resulted in a clinical consultation. The results ( Table 2.2ac) indicate that the mother was more likely than the father to report that he had consulted a doctor for the condition, but she was far less likely than he was to report the condition if he had not visited a doctor. Consequently, the father was far more likely than the mother to report that he had this problem. Comparing reports for the same periods of time – for example, taking the period from birth to 8 months later the mother reports asthma or wheezing for 7.4% of the fathers compared with 13.8% reported by the fathers themselves.

**Table 2.2ac.** Prevalence (%) of asthma or wheezing in study fathers and if so whether they consulted a doctor.

Time asked	Variable name	N	Period covered	And consulted Dr.		Not at all
				Yes %	No %	%
*Paternal report*						
8m	PD028	6829	Since baby was born	1.6	12.2	86.2
21m	PE028	6151	Since baby was 8m	1.7	13.1	85.2
4y	PG1018	4930	In past year	3.0	17.6	79.4
5y	PH1023	4435	In past year	3.1	14.9	82.0
6y	PJ3023	4432	Since child’s 5 ^th^ birthday	2.7	13.8	83.4
9y	PM1023	3616	Since child’s 6 ^th^ birthday	5.2	17.1	77.7
12y	PQ1023	3214	In the last 2 years	4.8	16.4	78.8
*Maternal report*						
8m	F529	10615	Since baby was born	2.9	4.5	92.6
21m	G615	9012	Since baby was 8m	4.5	5.1	90.5
33m	H500	9026	Since baby was 18m	5.0	4.8	90.3
4y	J618	8427	In past year	5.1	4.9	90.0
6y	L6034	7551	Since child was 5	5.4	5.9	88.7
9y	P3034	6583	in last 2 years	6.4	5.8	87.8
11y	S3034	6996	Since child’s 10 ^th^ birthday	4.7	5.2	90.1

N = no. completed

*2.2b. Breathlessness*

On three occasions the fathers were asked whether they had experienced attacks of breathlessness and if so, how frequently within the previous two years ( Table 2.2b). There was little change in prevalence over the 12 years covered.

**Table 2.2b.** Frequency of attacks of breathlessness reported by study fathers in past two years.

Time asked	Variable name	N	Often %	Sometimes %	Not at all %
Pregnancy	PA226	8477	2.1	10.9	87.0
8 years	PL1116	3949	1.9	11.2	86.9
11 years	PP2076	3591	1.4	9.4	89.2

N = no. completed

*2.2c. Bronchitis*

Bronchitis is an inflammation of the lining of the bronchial tubes. It may be either acute or chronic. Acute bronchitis often develops from a cold or other respiratory infection and is common and usually resolves within 10 days. Chronic bronchitis is more serious; it consists of a constant irritation or inflammation of the lining of the bronchial tubes and is often due to smoking.

Both the study mothers and their partners (the study fathers) were asked to state whether the father had had bronchitis during seven specified periods of time – and to state whether a doctor had been consulted for the problem ( Table 2.2c). Similar patterns of reply occurred for each.

**Table 2.2c.** Prevalence (%) of bronchitis in study fathers and if so whether they consulted a doctor.

Time asked	Variable name	N	Period covered	And consulted Dr.		Not at all
				Yes %	No %	%
*Paternal report*						
8m	PD029	6830	Since baby was born	0.9	0.8	98.2
21m	PE029	6151	Since baby was 8m	1.3	0.8	97.9
4y	PG1019	4921	In past year	1.9	1.2	96.9
5y	PH1024	4429	In past year	1.9	1.0	97.0
6y	PJ3024	4431	Since child’s 5 ^th^ birthday	1.7	1.4	96.9
9y	PM1024	3613	Since child’s 6 ^th^ birthday	2.2	1.1	96.7
12y	PQ1024	3204	In the last 2 years	1.4	0.8	97.8
*Maternal report*						
8m	F523	10615	Since baby’s birth	1.0	0.7	98.4
21m	G609	8947	Since baby was 8m	2.0	0.8	97.1
33m	H494	9026	Since baby was 18m	2.1	1.2	96.7
4y	J612	8396	In past year	2.3	1.1	96.6
6y	L6028	7450	Since child was 5	2.5	1.2	96.3
9y	P3028	7261	Since child was 7	2.5	1.2	96.3
11y	S3028	6524	Since child’s 10 ^th^ birthday	1.8	0.7	97.5

N = no. completed

*2.2d. Cough*

During the same specified times, each parent was asked whether the study father had had a cough or cold. Not surprisingly, the proportions reporting positively were high, with overall rates depending on the length of time covered. Relatively few of those with coughs and/or colds had consulted a doctor ( Table 2.2da).

**Table 2.2da.** Prevalence (%) of cough and/or cold in study fathers and if so whether they consulted a doctor.

Time asked	Variable name	N	Period covered	Consulted Dr.		Not at all
				Yes %	No %	%
*Paternal report*						
8m	PD025	6825	Since baby was born	3.9	64.4	31.8
21m	PE025	6151	Since baby was 8m	5.5	72.7	21.9
4y	PG1015	5030	In past year	4.4	76.1	19.5
5y	PH1015	4460	In past year	5.9	71.5	22.5
6y	PJ3017	4432	Since child’s 5 ^th^ birthday	4.6	73.8	21.7
9y	PM1015	3644	Since child’s 6 ^th^ birthday	4.4	80.2	15.3
12y	PQ1015	3227	In the last 2 years	3.1	72.3	24.6
*Maternal report*						
8m	F521	10615	Since baby’s birth	4.1	64.3	31.6
21m	G607	9530	Since baby was 8m	6.1	78.3	15.6
33m	H492	9026	Since baby was 18m	4.8	78.7	16.5
4y	J610	8605	In past year	4.7	78.4	16.9
6y	L6026	8254	Since child was 5	4.1	73.9	22.0
9y	P3026	7801	In last 2 years	3.1	76.3	20.6
11y	S3026	6900	Since child’s 10 ^th^ birthday	2.8	70.3	26.9

N = no. completed

Timing of coughs was collected at three time points: during pregnancy, and at 8 and 11 years later. The fathers were asked whether they coughed often during the night and on waking ( Table 2.2db). There was a suggestion that the proportion of fathers reporting often coughing on waking reduced over time. There was no similar trend for often coughing during the night.

**Table 2.2db.** Frequency of coughing often at night and on waking reported by study fathers in the past two years.

Time	Variable	N	Often %	Sometimes %	Not at all %
*Cough often at night*					
Pregnancy	PA227	8491	1.8	21.5	76.7
8 years	PL1117	3961	1.9	21.9	76.2
11 years	PP2077	3598	1.2	22.1	76.7
*Cough often on waking*					
Pregnancy	PA228	8487	3.2	17.7	79.1
8 years	PL1118	3943	2.6	16.7	80.7
11 years	PP2078	3595	2.2	16.2	81.6

N = no. completed

*2.2e. Influenza*

Each parent was asked as to whether the father had had influenza in specific periods of time. The incidences reported were similar, both for consultation of a doctor and with no such consultation ( Table 2.2e).

**Table 2.2e.** Prevalence of influenza reported for study fathers and if so whether they consulted a doctor.

Time asked	Variable name	N	Period covered	And consulted Dr.		Not at all
				Yes %	No %	%
*Paternal report*						
8m	PD026	6830	Since baby was born	2.5	9.1	88.4
21m	PE027	6151	Since baby was 8m	4.2	13.9	81.8
4y	PG1017	4932	In past year	3.8	15.2	81.0
5y	PH1021	4393	In past year	4.6	13.8	81.5
6y	PJ3021	4426	Since child’s 5 ^th^ birthday	3.4	14.9	81.7
9y	PM1021	3608	Since child’s 6 ^th^ birthday	3.7	17.9	78.4
12y	PQ1021	3186	In the last 2 years	1.5	9.2	89.3
*Maternal report*						
8m	F522	10615	Since baby’s birth	2.7	9.9	87.4
21m	G608	9049	Since baby was 8m	4.6	16.5	78.9
33m	H493	9026	Since baby was 18m	4.1	15.6	80.3
4y	J611	8443	In past year	3.8	17.0	79.2
6y	L6027	7632	Since child was 5	3.3	15.5	81.2
9y	P3027	7426	In last 2 years	2.9	16.3	80.8
11y	S3027	6551	Since child’s 10 ^th^ birthday	1.6	9.6	88.8

N = no. completed

***2.3. Hay fever and other allergies***

Hay fever, also called allergic rhinitis, causes cold-like symptoms which often include a runny nose, itchy eyes, congestion, sneezing and sinus pressure. It is caused by an allergic response to an airborne allergen such as dust or pollen.

Fathers were asked on three occasions whether they had hay fever in the past year or if not then, in the past but not the previous year; approximately a third responded positively ( Table 2.3aa). At the same times, they were asked whether they had experienced three common symptoms of hay fever: frequent sneezing attacks, runny nose and watery eyes. Approximately 30% reported sneezing attacks, 60% frequent runny nose, and a third frequent watery eyes. Reports of often having these three signs reduced over time ( Table 2.3ab).

**Table 2.3aa.** History of whether father had ever had hay fever distinguishing between recently (in past year) and prior to the past year only.

Time asked	Variable name	N	Yes		No
			Recently %	In past %	Never %
Pregnancy	PA170	8337	20.7	9.9	69.3
8 years	PL1040	3979	25.9	11.6	62.5
11 years	PP2000	3623	23.2	13.3	63.5

N = no. completed

**Table 2.3ab.** Frequency with which fathers reported that they experienced frequent sneezing, runny nose and watery eyes in the past two years.

Time asked	Variable name	N	Often %	Sometimes %	Not at all %
*Sneezing attacks*					
Pregnancy	PA223	8490	6.3	23.0	70.7
8 years	PL1113	3957	4.9	26.4	68.7
11 years	PP2073	3596	4.1	29.6	66.3
*Runny nose*					
Pregnancy	PA1224	8506	6.8	55.7	37.5
8 years	PL1114	3965	5.3	62.0	32.7
11 years	PP2074	3608	4.5	60.1	35.4
*Watery eyes*					
Pregnancy	PA225	8478	4.9	32.9	62.2
8 years	PL1115	3939	4.0	34.7	61.3
11 years	PP2075	3599	3.6	36.0	60.5

N = no. completed

*Specific allergies*

Allergies occur when the body's immune system reacts to a particular substance as though it's harmful. The most common allergens are pollen (especially to trees and grasses), mould, dust mites, pets (especially cats and dogs), and a variety of foods (especially nuts and peanuts). Individuals with allergies often also have asthma and eczema.

The father was asked about allergies on the same three occasions as for the signs of hay fever ( Table 2.3b). This indicated that about a third reported having an allergy, with pollen and dust being the most common. It should be noted that only 4–5 specific allergens were enquired, but there was an opportunity for the father to indicate ‘other’ and write in other allergens. The relevant text descriptions are available for interested scientists to code.

**Table 2.3b.** Proportion of fathers reporting ever having an allergy.

Allergy	Pregnancy		By 8 years		By 11 years	
	Variable name	% (n)	Variable name	% (n)	Variable name	% (n)
**Any**	PA210	35.1 (2954)	PL1100	34.8 (1356)	PP2060	34.5 (1256)
**Cat**	PA211	7.8 (662)	PL1101	10.1 (409)	PP2061	9.3 (340)
**Pollen**	PA212	16.9 (1434)	PL1102	18.6 (754)	PP2062	18.8 (684)
**Dust**	PA213	13.7 (1165)	PL1103	13.6 (551)	PP2063	13.7 (500)
**Insect bite**	PA214	3.2 (267)	PL1104	3.1 (125)	PP2064	4.3 (155)
**Medication**	-	-	PL1105	6.6 (269)	PP2065	6.8 (246)
**Other**	PA215	13.8 (1166)	PL1106	9.9 (402)	PP2066	9.2 (336)

n = no. with the allergy; - = not asked

***2.4. Skin conditions***

*2.4a Eczema*

Eczema, also known as atopic dermatitis, is a condition that makes the skin red and itchy. It is common in children but can occur at any age. It is long lasting (chronic) and tends to flare periodically. It may be accompanied by asthma or hay fever.

The father was asked on three occasions (during the study pregnancy and 8 and 11 years later) as to whether he had a history of eczema. Positive responses were received ranging from 13.5% during pregnancy to 20.8% eight years later ( Table 2.4aa). In parallel both the mothers and the fathers were asked about experience of eczema by the father ( Table 2.4ab). Answers from both parents showed similar rates, varying from approximately 6–8% stating that the father had had eczema in the relevant period of time.

**Table 2.4aa.** History of eczema in study fathers distinguishing between recently (in past year) and prior to the past year only.

Time asked	Variable name	N	Yes		No
			Recently %	In past %	Never %
Pregnancy	PA174	8392	6.5	7.0	86.5
8 years	PL1044	3967	10.0	10.8	79.2
11 years	PP2004	3624	9.1	10.0	80.9

N = no. completed

**Table 2.4ab.** Prevalence (%) of eczema in study fathers and if so whether they consulted a doctor.

Time asked	Variable name	N	Period covered	And consulted Dr		Not at all
				Yes %	No %	%
*Paternal report*						
8m	PD031	6829	Since baby was born	1.4	4.5	94.0
21m	PE031	6151	Since baby was 8m	2.1	5.1	92.9
4y	PG1021	4928	In past year	2.4	5.9	91.7
5y	PH1026	4428	In past year	1.8	5.7	92.5
6y	PJ3026	4429	Since child’s 5 ^th^ birthday	2.1	5.7	92.2
9y	PM1026	3614	Since child’s 6 ^th^ birthday	3.2	6.4	90.5
12y	PQ1026	3206	In the last 2 years	2.3	6.9	90.8
*Maternal report*						
8m	F530	10615	Since baby’s birth	1.4	3.4	95.2
21m	G616	8981	Since baby was 8m	2.5	3.8	93.7
33m	H501	9026	Since baby was 18m	2.5	4.1	93.4
4y	J619	8418	In past year	2.4	3.9	93.8
6y	L6035	7476	Since child was 5	2.0	4.4	93.6
9y	P3035	7291	In last 2 years	2.4	4.3	93.3
11y	S3035	6569	Since child’s 10 ^th^ birthday	1.8	4.4	93.8

N = no. completed

*2.4b. Psoriasis*

The skin disease psoriasis causes a rash with itchy, scaly patches, most commonly on the knees, elbows, trunk and scalp. It is a long-term (chronic) disease with no known cure. It can be painful, interfere with sleep and make it hard to concentrate. The condition tends to go through cycles, flaring for a few weeks or months, then subsiding for a while. Common triggers in people with a genetic predisposition to psoriasis include infections, cuts or burns, and certain medications.

Very few fathers reported psoriasis (3–5%); most of those who had had psoriasis reported that they had had the condition in the past year ( Table 2.4ba). A similar picture was seen when looking at the mothers’ and fathers’ reports for certain periods of time, with similar rates being reported by each parent. However, mothers reported that about half the affected fathers had consulted a doctor for the problem compared with less than a third of the fathers at each point in time ( Table 2.4bb).

**Table 2.4ba.** History of whether father had ever had psoriasis distinguishing between recently (in past year) and prior to the past year only.

Time asked	Variable Name	N	Yes		No
			Recently %	In past %	Never %
Pregnancy	PA184	8277	2.2	0.9	96.9
8 years	PL1054	3958	3.6	1.9	94.4
11 years	PP2014	3616	3.6	2.0	94.4

N = no. completed

**Table 2.4bb.** Prevalence (%) of psoriasis in study fathers and if so whether they consulted a doctor.

Time asked	Variable name	N	Period covered	And consulted Dr		Not at all
				Yes %	No %	%
*Paternal report*						
8m	PD032	6830	Since baby was born	0.7	1.4	97.9
21m	PE032	6151	Since baby was 8m	0.9	1.4	97.7
4y	PG1022	3925	In past year	1.2	2.2	96.6
5y	PH1027	4404	In past year	1.2	2.5	96.3
6y	PJ3027	4430	Since child’s 5 ^th^ birthday	1.5	2.5	96.1
9y	PM1027	3618	Since child’s 6 ^th^ birthday	1.8	2.4	95.8
12y	PQ1027	3200	In the last 2 years	1.4	2.3	96.3
*Maternal report*						
8m	F531	10615	Since baby’s birth	0.7	1.1	98.1
21m	G617	8967	Since baby was 8m	1.2	1.4	97.4
33m	H502	9026	Since baby was 18m	1.4	1.5	97.0
4y	J620	8410	In past year	1.5	1.6	96.8
6y	L6036	7474	Since child was 5	1.8	1.8	96.4
9y	P3036	7264	In past 2 years	1.7	1.9	96.4
11y	S3036	6558	Since child’s 10th birthday	1.5	2.0	96.5

N = no. completed

*2.4c. Dermatitis*

Dermatitis is a general term that describes a common skin irritation. We have used it here to cover the answers to the description of a dry itchy rash. On three occasions the study fathers were asked how often they had had such a rash in the past two years. It can be seen from Table 2.4c that about a fifth of fathers reported that they had had such a rash, the majority of whom reported that they had had the rash sometimes rather than often.

**Table 2.4c.** Frequency of dermatitis (dry itchy rash) in past 2 years by study fathers.

Time asked	Variable name	N	Often %	Sometimes %	Not at all %
Pregnancy	PA221	8492	3.8	15.3	80.9
8 years	PL1111	3955	3.3	17.3	79.3
11 years	PP2071	3589	3.0	18.9	79.0

N = no. completed

*2.4d. Hives*

Hives (urticaria or nettle rash) is a skin reaction that causes itchy welts that range in size from small spots to large blotches. The reaction can be triggered by many situations and substances, including stress, certain foods and medications.

Immediately after the question on the dry itchy rash, the study fathers were asked whether they had had ‘a blotchy blistery rash (hives) in the past two years’. There were very similar responses on each of the three occasions with about 3% saying that they had had such a rash. Only 0.4-0.5% claimed to have such a rash often, and the remaining 3% had stated they had had it sometimes ( Table 2.4d).

**Table 2.4d.** Frequency of hives in past 2 years by study fathers.

Time asked	Variable name	N	Often %	Sometimes %	Not at all %
Pregnancy	PA222	8465	0.5	2.8	96.7
8 years	PL1112	3932	0.5	2.7	96.8
11 years	PP2072	3579	0.4	3.2	96.5

N = no. completed

***2.5 Gastrointestinal problems***

*2.5a. Indigestion*

Indigestion describes certain symptoms, such as abdominal pain and a feeling of fullness soon after starting eating, rather than a specific disease. However, it can also be a symptom of various digestive diseases. It was a relatively common problem among the fathers in the study and experienced by 27–41% within the previous year ( Table 2.5aa). Less than 1% of the fathers, however, claimed to have had indigestion continuously in the past month ( Table 2.5ab).

**Table 2.5aa.** History of whether father had ever had indigestion distinguishing between recently (in past year) and prior to the past year.

Time asked	Variable name	N	Yes		No
			Recently %	In past %	Never %
Pregnancy	PA171	8395	26.6	36.9	36.5
8 years	PL1041	3988	41.0	34.2	24.8
11 years	PP2001	3625	35.1	41.8	23.1

N = no. completed

**Table 2.5ab.** Frequency (%) of indigestion in study fathers in the past month.

Time asked	Variable Name	N	Always %	Sometimes %	Not at all %
21m	PE123	6140	0.6	23.9	75.5
4y	PG1143	5034	0.7	29.1	70.1
5y	PH1163	4474	0.6	29.9	69.5
6y	PJ3253	4441	0.7	30.6	68.7
9y	PM1203	3648	0.7	29.8	69.5
11y	PP2153	3624	1.4	37.4	61.1
12y	PQ1203	3247	0.6	30.1	69.3

N = no. completed; *Question included an option ‘once only’. This has been combined with ‘sometimes’

Both parents recorded whether the father had had indigestion and, if so, had consulted a doctor during 7 specific periods of time. Although 30–40% of fathers were reported to have the condition, in general <4% of the fathers had consulted a doctor for the problem ( Table 2.5ac).

**Table 2.5ac.** Prevalence (%) of indigestion in study fathers and if so whether they consulted a doctor.

Time asked	Variable name	N	Period covered	And consulted Dr		Not at all
				Yes %	No %	%
*Paternal report*						
8m	PD024	6828	Since baby was born	1.4	25.8	72.8
21m	PE024	6151	Since baby was 8m	1.7	28.0	70.4
4y	PG1014	4951	In past year	2.6	35.3	62.1
5y	PH1014	4411	In past year	2.4	35.7	61.8
6y	PJ3015	4426	Since child’s 5th birthday	2.6	37.0	60.3
9y	PM1015	3617	Since child’s 6th birthday	3.7	42.0	54.4
12y	PQ1015	3210	In the last 2 years	3.7	39.7	56.6
*Maternal report*						
8m	F516	10615	Since baby’s birth	1.6	22.4	76.0
21m	G602	8802	Since baby was 8m	2.5	27.9	69.5
33m	H487	9026	Since baby was 18m	3.1	28.8	68.1
4y	J605	8196	In past year	3.1	30.6	66.3
6y	L6021	7549	Since child was 5	3.2	38.8	58.0
9y	P3021	7273	In last 2 years	4.6	36.8	58.6
11y	S3021	6460	Since child’s 10th birthday	3.5	38.2	58.3

N = no. completed

*2.5b. Haemorrhoids*

Haemorrhoids, or piles, are painful swollen veins in the anus and lower rectum, similar to varicose veins. Haemorrhoids were common in our study, with the proportion of fathers reporting they had ever had the problem increasing from 20% at the time of pregnancy to 30% after 8 – 11 years ( Table 2.5ba). The frequency with which they had occurred during the past month was asked on 7 occasions. They indicated a slight increase over time, from 7.3% at 21 months to 10.0% ten years later; relatively few fathers reported that the haemorrhoids were always present ( Table 2.5bb). Mothers’ reports of the fathers’ haemorrhoids were proportionately very similar to those of the father himself ( Table 2.5bc).

**Table 2.5ba.** History of whether father had ever had haemorrhoids (piles) distinguishing between recently (in past year) and prior to the past year only.

Time asked	Variable name	N	Yes		No Never %
			Recently %	In past %	
Pregnancy	PA181	8458	7.0	13.5	79.5
8 years	PL1051	3975	13.0	18.6	68.4
11 years	PP2011	3616	11.4	19.0	69.6

N = no. completed

**Table 2.5bb.** Frequency (%) of haemorrhoids in study fathers in the past month.

Time asked	Variable name	N	Always %	Sometimes %	Not at all %
21m *	PE116	6140	0.8	6.5	92.7
4y	PG1136	4970	1.2	7.7	91.1
5y *	PH1156	4283	1.1	7.2	91.7
6y	PJ3246	4302	1.6	8.0	90.4
9y	PM1196	3630	1.5	8.3	90.2
11y	PP2146	3609	2.2	10.6	87.3
12y	PQ1196	3238	1.2	8.8	90.0

N = no. completed; *Question included an option ‘once only’. This has been combined with ‘sometimes’

**Table 2.5bc.** Prevalence (%) of haemorrhoids in study fathers and if so whether they consulted a doctor.

Time asked	Variable name	N	Period covered	And consulted Dr.		Not at all
				Yes %	No %	%
*Paternal report*						
8m	PD027	6828	Since baby was born	1.2	7.0	91.8
21m	PE026	6151	Since baby was 8m	1.5	8.5	90.0
4y	PG1016	4927	In past year	1.8	11.9	86.3
5y	PH1019	4390	In past year	2.3	9.8	87.9
6y	PJ3019	4427	Since child’s 5 ^th^ birthday	2.5	11.2	86.2
9y	PM1019	3611	Since child’s 6 ^th^ birthday	4.7	13.0	82.3
12y	PQ1019	3190	In the last 2 years	2.7	13.6	83.7
*Maternal report*						
8m	F520	10615	Since baby’s birth	1.4	6.4	92.3
21m	G606	8828	Since baby was 8m	2.1	8.1	89.8
33m	H491	9026	Since baby was 18m	2.8	9.4	87.8
4y	J609	8212	In past year	2.6	9.4	88.1
6y	L6025	7314	Since child was 5	2.7	10.4	86.9
9y	P3025	7110	In last 2 years	3.6	10.7	85.7
11y	S3025	6262	Since child’s 10 ^th^ birthday	2.6	10.7	86.7

N = no. completed

*2.5c. Stomach ulcer*

Stomach or peptic ulcers are open sores that develop on the inside lining of the stomach (gastric ulcers) and the upper portion of the small intestine (duodenal ulcers). The most common symptom of which is stomach pain. Only 3-4% of the study fathers reported ever having had such a disorder, and no more than 1% had had it in the previous year when asked in pregnancy, a proportion that was similar 8 and 11 years later ( Table 2.5ca). Study mothers appeared to be more likely to know of the disorder in their partners if they had consulted a doctor for the problem ( Table 2.5cb).

**Table 2.5ca.** History of whether father had ever had a stomach ulcer distinguishing between recently (in past year) and prior to the past year only.

Time asked	Variable name	N	Yes		No
			Recently %	In past %	Never %
Pregnancy	PA185	8395	0.8	2.5	96.6
8 years	PL1055	3968	1.0	3.2	95.8
11 years	PP2015	3621	0.6	2.9	96.5

N = no. completed

**Table 2.5cb.** Prevalence (%) of stomach ulcer in study fathers and if so whether they consulted a doctor.

Time asked	Variable name	N	Period covered	And consulted Dr.		Not at all
				Yes %	No %	%
*Paternal report*						
8m	PD030	6829	Since baby was born	0.5	0.3	99.2
21m	PE030	6151	Since baby was 8m	0.6	0.2	99.2
4y	PG1020	4915	In past year	0.7	0.4	98.9
5y	PH1025	4426	In past year	0.6	0.5	98.9
6y	PJ3025	4426	Since child’s 5 ^th^ birthday	0.9	0.5	98.7
9y	PM1025	3614	Since child’s 6 ^th^ birthday	1.0	0.2	98.8
12y	PQ1025	3203	In the last 2 years	0.6	0.2	99.2
*Maternal report*						
8m	F528	10615	Since baby’s birth	0.8	0.4	98.8
21m	G614	8939	Since baby was 8m	1.1	0.5	98.5
33m	H499	9026	Since baby was 18m	1.2	0.5	98.3
4y	J617	8381	In past year	1.2	0.5	98.3
6y	L6033	7426	Since child was 5	1.3	0.7	98.0
9y	P3033	7247	In past 2 years	1.4	0.5	98.1
11y	S3033	6533	Since child’s 10 ^th^ birthday	0.9	0.6	98.5

N = no. completed

*2.5d. Diarrhoea*

Diarrhoea is generally defined as loose, watery and possibly more-frequent bowel movements. It is usually short-lived, lasting no more than a few days. But it can last into weeks; if so, it usually indicates that there is another problem. Our study fathers reported on seven occasions as to how frequently they had had diarrhoea in the preceding month. Very few ((<0.5%) recorded that this was occurring throughout the month ( Table 2.5d).

**Table 2.5d.** Frequency (%) of diarrhoea in study fathers in the past month.

Time asked	Variable name	N	Always	Sometimes	Not at all
21m	PE115	6140	< 0.1	16.3	83.6
4y	PG1135	4968	< 0.2	13.1	86.8
5y	PH1155	4287	0.2	16.6	83.2
6y	PJ3245	4302	0.3	13.0	86.8
9y	PM1195	3627	0.3	13.0	86.7
11y	PP2145	3613	0.5	19.7	79.8
12y	PQ1195	3237	0.4	15.3	84.3

N = no. completed; *Question included an option ‘once only’. This has been combined with ‘sometimes’

*2.5e. Nausea and Vomiting*

Nausea and vomiting are common signs and symptoms that can be caused by numerous conditions. Although they are often due to viral gastroenteritis, many medications or substances can also cause nausea and vomiting, including marijuana (cannabis).

Fathers were asked about the frequency with which they had had each of the two symptoms in the previous month ( Table 2.5e). Very few men (<0.2%) stated that they had such problems throughout the month, but 5–8% had nausea and 3–7% had vomited at some time during the month.

**Table 2.5e.** Frequency (%) of nausea and vomiting in study fathers in the past month.

Time asked	Variable name	N	Always	Sometimes	Not at all
*Nausea*					
21m	PE113	6140	< 0.2	7.5	92.4
4y	PG1133	4969	< 0.2	5.6	94.3
5y	PH1153	4273	< 0.2	7.3	92.7
6y	PJ3243	4302	< 0.2	5.4	94.6
9y	PM1193	3630	< 0.2	5.1	94.8
11y	PP2143	3328	< 0.2	7.8	92.1
12y	PQ1193	3236	< 0.2	5.4	94.5
*Vomiting*					
21m	PE114	6140	< 0.2	6.9	93.1
4y	PG1134	4967	< 0.2	4.5	95.5
5y	PH1154	4273	< 0.2	7.3	92.7
6y	PJ3244	4301	< 0.2	3.7	96.3
9y	PM1194	3624	< 0.2	3.3	96.6
11y	PP2144	3612	< 0.2	6.0	93.9
12y	PQ1194	3237	< 0.2	4.3	95.7

N = no. completed *Question included an option ‘once only’. This has been combined with ‘sometimes’

***2.6 Cardiovascular features***

*2.6a. Hypertension*

Hypertension is the name given to the presence of high blood pressure. This is not a disease in itself but increases the risk of heart disease and stroke. The prevalence is known to increase with age.

There were two occasions when the father was asked whether he had ever had hypertension, the age at which he had first had it, and whether he currently had it. The results are shown in Table 2.6aa. Eight years after the child’s birth, 5.9% of the fathers reported that they had had high blood pressure; three years later this had increased to 9.0%. Similarly, the point prevalence had increased from 3.1% to 4.7%. On both occasions the modal age at which hypertension was reported to have developed was 40; however, it should be noted that there was marked numerical bias with multiples of ten being particularly reported.

**Table 2.6aa.** History of hypertension as recorded on two occasions: at 8 and 11 years after the birth of the study child.

Time asked	Variable name	N	Measure	Result
8y	PL1080	4047	Ever had	5.9% yes
	PL1082	4047	Age first had	Mode 40y
	PL1084	4047	Has it now	3.1% yes
11y	PP2040	3640	Ever had	9.0% yes
	PP2042	3640	Age first had	Mode 40y
	PP2043	3640	Has it now	4.7% yes

N = no. completed

ALSPAC questionnaires also asked the father whether he had had high blood pressure (not specified) / hypertension within 4 specific times, but the mother was asked whether her partner had the problem on 7 occasions ( Table 2.6ab). Both parents’ reports indicated that the proportion of fathers with hypertension had increased by the time the study children were aged 9 or more.

**Table 2.6ab.** Prevalence (%) of high blood pressure/hypertension in study fathers within certain periods of time and whether they consulted a doctor.

Time asked	Variable name	N	Period covered	And consulted Dr.		Not at all
				Yes %	No %	%
*Paternal report*						
5y	PH1016	4355	In past year	2.4	1.3	96.3
6y	PJ3016	4419	Since child’s 5 ^th^ birthday	2.2	1.8	96.0
9y	PM1016	3599	Since child’s 6 ^th^ birthday	5.6	1.8	92.6
12y	PQ1016	3186	In the last 2 years	8.7	2.2	89.1
*Maternal report*						
8m	F524	10615	Since baby’s birth	1.4	0.7	98.0
21m	G610	8758	Since baby was 8m	1.9	0.8	97.3
33m	H495	9026	Since baby was 18m	2.1	0.8	97.0
4y	J613	8134	In past year	2.4	0.8	96.9
6y	L6029	7215	Since child was 5	3.2	0.8	96.0
9y	P3029	7058	In last 2 years	7.1	0.9	92.0
11y	S3029	6312	Since child’s 10th birthday	8.4	1.5	90.1

N = no. completed

*2.6b. Chest pain*

Chest pain is reported to appear in many forms, ranging from a sharp stab to a dull ache. It can be caused by many different conditions, the most notable being angina – a symptom of coronary heart disease caused by reduced blood flow to the heart. The questions asked of the parents in ALSPAC comprised those developed by Geoffrey Rose and colleagues ( Rose *et al.*, 1977). They used the questions to distinguish between angina, possible infarction and intermittent claudication. After using it on over 18,000 men they stated that the self-administered version of their questionnaire provided a simple and convenient means of identifying individuals with a high risk of major coronary heart disease.

The Rose questions were asked of the father at two time points (7 and 11 years after the study child was born). Similar proportions of the fathers reported ever having chest pain (25.8% and 26.2%) at the two time points ( Table 2.6b). Further details as to the activities associated with the pain, severity of the pain and the number of occurrences can be obtained from the relevant ‘built file’ documentation in the ALSPAC Data Dictionary. It should be noted that the situation of the pain within the chest was marked on a diagram of the chest, and coded. Text descriptions of the diagnosis that had been given to the father are available, and the three different conditions can be derived from the questions using the criteria described in the paper by Rose and his colleagues.

**Table 2.6b.** Responses to questions concerning chest pain asked of the study fathers 7 years (PK) and 11 years (PP) after the study child was born.

Time asked	Variable name	N	Yes in past year %	Yes but not in past year %	No not at all %
7 years	PK4040	3960	16.0	9.8	74.2
11 years	PP2120	3615	13.8	12.4	73.8

N = no. completed

*2.6c. Diabetes*

Diabetes mellitus refers to a group of diseases that affect the body’s relationship with blood sugar (glucose). Glucose is vital to health as an important source of energy for muscles and tissues. It is the brain’s main source of fuel. Consequently, diabetes can have major effects on health and well-being. There are two types of diabetes – both can lead to excess sugar in the blood and can lead to serious health problems. Type 1 diabetes is the type that requires daily insulin to stabilise it and often develops in childhood, and type 2, which rarely occurs in childhood, can usually be controlled by diet and/or oral medication.

Both parents were asked whether the father had diabetes. According to the mother only 0.3% of the fathers had diabetes shortly after the birth of the study child, but 11 years later the proportion had increased six-fold to 1.9%. The father was not asked directly about diabetes until the child was 5 years old, at which time 0.5% stated they were diabetic; 7 years later the prevalence had increased three-fold to 1.6% ( Table 2.6ca). 

**Table 2.6ca.** Prevalence (%) of diabetes in study fathers and if so whether they consulted a doctor.

Time asked	Variable name	N	Period covered	And consulted Dr.		Not at all
				Yes %	No %	%
*Paternal report*						
5y	PH1018	4366	In past year	0.5	< 0.2	99.5
6y	PJ3018	4426	Since child’s 5 ^th^ birthday	0.5	< 0.2	99.3
9y	PM1018	3596	Since child’s 6 ^th^ birthday	0.8	< 0.2	99.1
12y	PQ1018	3177	In the last 2 years	1.4	< 0.2	98.4
*Maternal report*						
8m	F525	10615	Since baby’s birth	0.3	< 0.2	99.7
21m	G611	8953	Since baby was 8m	0.4	< 0.2	99.6
33m	H496	9026	Since baby was 18m	0.4	< 0.2	99.6
4y	J614	8404	In past year	0.4	< 0.2	99.5
6y	L6030	7441	Since child was 5	0.6	< 0.2	99.3
9y	P3030	7455	Since child was 7	1.0	< 0.2	98.9
11y	S3030	6536	Since child’s 10 ^th^ birthday	1.7	< 0.2	98.1

N = no. completed

*2.6d. Varicose veins*

Varicose veins are twisted, enlarged veins, most commonly affecting the veins in the legs. For many people, varicose veins are simply a cosmetic problem, but for others they can cause aching pain and discomfort.

The study fathers had an increasing prevalence of varicose veins with time – from 2.8% during the index pregnancy to 5.8% 11 years later ( Table 2.6da). Additional data collected on 7 occasions concerned the frequency with which their varicose veins were present in the preceding month. These indicate that as the father got older, varicose veins became reported more frequently ( Table 2.6db).

**Table 2.6da.** History of whether father had ever had varicose veins distinguishing between recently (in past year) and prior to the past year.

Time asked	Variable name	N	Yes		No
			Recently %	In past %	Never %
Pregnancy	PA180	8503	2.1	0.7	97.2
8 years	PL1050	3771	3.6	1.3	95.1
11 years	PP2010	3618	3.7	2.1	94.2

N = no. completed

**Table 2.6db.** Frequency (%) of varicose veins in study fathers in the past month.

Time asked	Variable name	N	Always	Sometimes	Not at all
21m *	PE120	6140	0.7	0.5	98.7
4y	PG1140	4871	0.8	0.8	98.3
5y *	PH1160	4259	1.0	1.1	98.0
6y	PJ3250	4434	0.8	1.4	97.8
9y	PM1200	3635	1.2	1.7	97.1
11y	PP2150	3611	2.0	1.9	96.1
12y	PQ1200	3239	1.9	1.6	96.5

N = no. completed; *Question included an option ‘once only’. This has been combined with ‘sometimes’

***2.7. Renal conditions***

*2.7a. Kidney disease*

Chronic kidney disease, also known as chronic kidney failure, involves a gradual loss of kidney function. Since the kidneys filter waste and excess fluids from the blood, which are then excreted in the urine, chronic kidney disease can cause dangerous levels of fluid, electrolytes and wastes to build up in the body. The fathers were asked on three occasions as to whether they had ever had kidney disease. Overall, there was little suggestion that the prevalence of such a history had varied from the 2.1% reported at the time of the study pregnancy ( Table 2.7a).

**Table 2.7a.** History of whether father had ever had kidney disease distinguishing between recently (in past year) and prior to the past year only.

Time asked	Variable name	N	Yes		No
			Recently %	In past %	Never %
Pregnancy	PA179	8492	0.3	1.8	97.9
8 years	PL10	3961	0.7	1.9	97.4
11 years	PP2009	3624	0.7	1.9	97.4

N = no. completed

*2.7b. Urinary infection*

Urinary tract infections encompass infections in any part of the urinary system (i.e. kidneys, ureters, bladder and urethra). However, most urinary infections involve the lower urinary tract (the bladder and urethra). Although women are prone to frequent urinary infections, it can be seen from Table 2.7ba and Table 2.7bb that when the preceding month was considered, the prevalence rarely exceeded 1% of fathers – and even when more extended periods of time were considered, only rarely did the condition occur as often as in 3% of fathers.

**Table 2.7ba.** Frequency (%) of urinary infection in study fathers in the past month.

Time asked	Variable name	N	Always/ Sometimes %	Not at all %
21m	PE112	6140	1.0	99.0
4y	PG1132	4966	0.8	99.2
5y	PH1152	4274	0.8	99.2
6y	PJ3242	4301	0.8	99.2
9y	PM1192	3626	0.5	99..5
11y	PP2142	3615	1.4	98.6
12y	PQ1192	3237	0.6	99.4

N = no. completed; *Question included an option ‘once only’. This has been combined with ‘sometimes’

**Table 2.7bb.** Prevalence (%) of urinary infection in study fathers and if so whether they consulted a doctor.

Time asked	Variable Name	N	Period covered	And consulted Dr.		Not at all
				Yes %	No %	%
*Paternal report*						
8m	PD035	6830	Since baby was born	0.9	0.4	98.6
21m	PE035	6151	Since baby was 8m	1.4	0.5	98.1
4y	PG1025	4909	In past year	1.4	0.7	97.9
5y	PH1030	4425	In past year	1.3	0.7	97.9
6y	PJ3030	4430	Since child’s 5 ^th^ birthday	1.7	0.6	97.7
9y	PM1030	3614	Since child’s 6 ^th^ birthday	2.3	1.0	96.7
12y	PQ1030	3207	In the last 2 years	2.0	0.7	97.3
*Maternal report*						
8m	F533	10615	Since baby’s birth	0.7	0.3	99.0
21m	G619	8931	Since baby was 8m	1.1	0.2	98.6
33m	H504	9026	Since baby was 18m	1.4	0.3	983
4y	J622	8387	In past year	1.2	0.3	98.5
6y	L6038	7421	Since child was 5	1.4	0.3	98.3
9y	P3038	7233	In past 2 years	1.8	0.4	97.8
11y	S3038	6516	Since child’s 10 ^th^ birthday	1.4	0.3	98.3

N = no. completed

**3. Psychiatric and Neurological conditions**

***3.1. Psychiatric disorders***

*3.1a. Addictions*

Substance addiction, also called substance use disorder, affects a person’s brain and behaviour and leads to an inability to control the use of the drug or medication. Substances such as alcohol, marijuana and nicotine are considered such drugs. Few of the fathers reported that they had had a drug addiction (by which they may have interpreted the question to refer to illicit drugs only), and the proportion was higher at the time of the pregnancy (1.2%) than 8–11 years later (0.4% – 0.5%) ( Table 3.1aa).

**Table 3.1aa.** History of whether father had ever had a drug addiction distinguishing between recently (in past year) and prior to the past year only.

Time asked	Variable	N	Yes		No
			Recently %	In past %	Never %
Pregnancy	PA187	8491	0.4	0.8	98.8
8 years	PL1057	3948	< 0.2	0.4	99.5
11 years	PP2017	3626	< 0.2	0.3	99.6

N = no. completed

Alcoholism, however, appeared to be more prevalent ( Table 3.1ab); again, the reported prevalence was higher during pregnancy (2.9%) than later (1.5%). Information was collected on several occasions from both the mother and father. Of those who answered the questions the mothers reported a higher prevalence than the fathers did concerning their own alcohol problems. It is notable also that very few of the men with alcohol problems had consulted a doctor ( Table 3.1ac). (It is worth noting that there were questions throughout that identified the amount of alcohol being consumed by the study father, which were answered by both the mother and her partner).

**Table 3.1ab.** History of whether father had ever had alcoholism distinguishing between recently (in past year) and prior to the past year.

Time asked	Variable name	N	Yes		No
			Recently %	In past %	Never %
Pregnancy	PA188	8429	0.4	2.5	97.1
8 years	PL1058	3968	0.3	1.2	98.5
11 years	PP2018	3624	0.7	0.8	98.5

N = no. completed

**Table 3.1ac.** Prevalence (%) of alcohol problems in study fathers and if so whether they consulted a doctor.

Time asked	Variable name	N	Period covered	And consulted Dr.		Not at all
				Yes %	No %	
*Paternal report*						
5y	PH1022	4429	In past year	0.2	1.2	98.6
6y	PJ3022	4435	Since child’s 5 ^th^ birthday	0.2	1.8	98.0
9y	PM1022	3620	Since child’s 6 ^th^ birthday	0.4	1.4	98.2
12y	PQ1022	3207	In the last 2 years	0.3	1.4	98.3
*Maternal report*						
8m	F527	10615	Since baby’s birth	0.2	0.9	98.9
21m	G613	8972	Since baby was 8m	0.3	1.6	98.0
33m	H498	9026	Since baby was 18m	0.3	2.1	97.6
4y	J616	8394	In past year	0.3	1.7	97.9
6y	L6032	7885	Since child was 5	0.3	2.2	97.5
9y	P3032	6353	In past 2 years	0.7	2.3	97.0
11y	S3032	6550	Since child’s 10 ^th^ birthday	0.5	2.4	97.1

N = no. completed

*3.1b. Depression*

Depression is a mood disorder that causes a persistent feeling of sadness and loss of interest; it affects the way people feel, think and behave and can lead to a variety of emotional and physical problems. Data were collected from ALSPAC parents in two ways: (i) direct questioning concerning whether they felt depressed, and (ii) using a validated set of questions which have been used to develop a score, high levels of which have been shown to predict clinical levels of depression.

The direct questioning method was used with three different types of question: first, the father was asked on three occasions concerning severe depression ever, distinguishing between the preceding year, and prior to that ( Table 3.1ba) – the proportions with severe depression were about 6% of fathers, the majority reporting previous episodes rather than recent ones; second, both mothers and fathers were asked concerning the presence of depression during a defined period of time, distinguishing between consulting a doctor or not ( Table 3.1bb) – this showed a higher reported prevalence, particularly a higher proportion consulting a doctor; third, the point prevalence concerning the frequency of depression in the preceding month was reported ( Table 3.1bc).

**Table 3.1ba.** History of whether father had ever had severe depression distinguishing between recently (in past year) and prior to the past year only.

Time asked	Variable name	N	Yes		No
			Recently %	In past %	Never %
Pregnancy	PA191	8428	0.8	5.4	93.9
8 years	PL1061	3961	1.5	4.3	94.2
11 years	PP2021	3617	1.6	4.7	93.8

N = no. completed

**Table 3.1bb.** Prevalence (%) of depression in study fathers and if so whether they consulted a doctor.

Time asked	Variable name	N	Period covered	And consulted Dr.		Not at all
				Yes %	No %	%
*Paternal report*						
8m	PD021	6828	Since baby was born	1.1	9.6	89.3
21m	PE021	6151	Since baby was 8m	1.5	9.9	88.6
4y	PG1011	4933	In past year	2.6	11.4	86.0
5y	PH1011	4391	In past year	3.0	9.8	87.3
6y	PJ3011	4427	Since child’s 5 ^th^ birthday	3.2	10.0	86.9
9y	PM1011	3607	Since child’s 6 ^th^ birthday	5.4	9.3	85.3
12y	PQ1011	3202	In the last 2 years	5.7	9.3	84.9
*Maternal report*						
8m	F518	10615	Since baby’s birth	1.2	6.8	92.0
21m	G604	8786	Since baby was 8m	1.8	8.6	89.6
33m	H489	9026	Since baby was 18m	2.3	9.2	88.5
4y	J607	8264	In past year	2.8	9.2	88.0
6y	L6023	7378	Since child was 5	3.9	10.3	85.8
9y	P3023	7165	In past 2 years	5.0	9.4	85.6
11y	S3023	6378	Since child’s 10 ^th^ birthday	5.6	10.2	84.2

N = no. completed

**Table 3.1bc.** Frequency (%) of depression in study fathers in the past month.

Time asked	Variable name	N	Always %	Sometimes %	Not at all %
21m *	PE130	6140	0.5	16.9	82.5
4y	PG1150	5026	0.6	16.5	82.9
5y *	PH1170	4453	0.6	18.7	80.7
6y	PJ3260	4443	1.1	18.7	80.2
9y	PM1210	3639	1.0	15.7	83.2
11y	PP2160	3620	1.0	18.1	80.9
12y	PQ1210	3230	0.9	16.8	82.2

N = no. completed; *Question included an option ‘once only’. This has been combined with ‘sometimes’

The Edinburgh Postnatal Depression Scale (EPDS; Cox *et al.*, 1987) was selected by ALSPAC to be used for identifying depression among pregnant women. Others have shown that it is a valid instrument for use in men ( Shafian *et al.*, 2022). It has been shown that, using a score of >12 to identify depression in ALSPAC parents postnatally identified 10% of mothers but only 4% of fathers as depressed ( Ramchandani *et al.*, 2005). Others have used lower cut-points (e.g. 10+ by Hibbeln *et al.*, 2018). Massoudi *et al.* (2013) carried out a detailed validation study on men in Sweden and concluded that the EPDS is ‘a valid instrument for screening for probable major depression, but it is questionable if it should be used to screen for minor depression.’ More recently Shafian and colleagues (2022) carried out a systematic review and showed that cut points of 7–10 for fathers identified depression satisfactorily. Over the 12 years covered by this Data Note the scale was measured on 8 occasions from mid-pregnancy to 11 years after delivery. Table 3.1bd identifies the EPDS scores at the different time points and gives the proportions with scores exceeding 9 and 12. Details of the variables comprising the scale are given in the Data Note by Paul and Pearson (2020).

**Table 3.1bd.** Continuous measures of the EPDS to assess current depression scores with proportions scoring over 9 and over 12.

Time	Variable	N	% >9	% >12
18wk gestation	PB260	9722	10.6	4.1
2 months	PC102	8235	9.0	3.5
8 months	PD200	6997	7.3	2.9
21 months	PE290	6098	8.4	3.5
33 months	PF4140	5325	9.1	3.8
6 years	PJ2103	4405	14.0	6.3
8 years	PL6100	3893	12.8	6.1
11 years	PP4053	3549	11.8	5.5

N = no. completed

*3.1c. Anxiety*

People with anxiety disorders frequently have intense, excessive and persistent worry and fear about everyday situations. Often, anxiety disorders involve repeated episodes of sudden feelings of intense anxiety and fear or terror that reach a peak within minutes (panic attacks). These feelings can interfere with daily activities, are difficult to control, are out of proportion to the actual danger and can last a long time.

As with depression, experiences of anxiety were obtained using two methods: direct enquiry as to experiences of anxiety, and scores on a scale of questions that rate current levels of anxiety. In regard to the direct questions, both the mother and the father were asked about the father’s experience of anxiety ( Table 3.1ca). This indicated that both reports were similar with mothers reporting prevalences that varied from 11% to 20% and fathers reporting from 14% to 22% at different time periods.

**Table 3.1ca.** Prevalence (%) of anxiety in study fathers and if so whether they consulted a doctor.

Time asked	Variable name	N	Period covered	And consulted Dr.		Not at all
				Yes %	No %	%
*Paternal report*						
8m	PD020	6830	Since baby was born	1.6	12.2	86.2
21m	PE020	6151	Since baby was 8m	1.7	13.1	85.2
4y	PG1010	3925	In past year	3.0	17.6	79.4
5y	PH1010	4404	In past year	3.1	14.9	82.0
6y	PJ3010	4430	Since child’s 5 ^th^ birthday	2.7	13.8	83.4
9y	PM1010	3618	Since child’s 6 ^th^ birthday	5.2	17.1	77.7
12y	PQ1010	3200	In the last 2 years	4.8	16.4	78.8
*Maternal report*						
8m	F519	10615	Since baby’s birth	1.3	10.4	88.3
21m	G605	8768	Since baby was 8m	1.7	13.5	84.8
33m	H490	9026	Since baby was 18m	2.0	14.0	84.0
4y	J608	8230	In past year	2.6	14.7	82.6
6y	L6024	7332	Since child was 5	2.9	16.9	80.2
9y	P3024	7132	In past 2 years	4.2	13.8	82.0
11y	S3024	6338	Since child’s 10 ^th^ birthday	3.9	15.1	81.0

The scale used to measure anxiety in both the mothers and fathers used the sub-scale of free-floating anxiety, which is part of the *Crown-Crisp Experiential Index* ( *CCEI;* Crown & Crisp, 1979). The original sub-scale had varying styles of response categories, some questions having a 2-point yes/no scale, while others had 3-point categories. ALSPAC modified the response categories for the study so that each item had 4 consistent response categories to which the respondent indicated frequency of symptoms from ‘never’ to ‘very often’. These modifications were extensively pilot tested including a validation study against the Present State Examination ( Thorpe, 1993). In a pilot study of a random sample of 54 pregnant women attending a routine check-up, Crown–Crisp index correlated 0.70 and 0.76 with the State and Trait (respectively) subscales of the Spielberger State-trait Anxiety Inventory ( Heron *et al.*, 2004). Over the 12 years covered by this Data Note the scale was measured on 8 occasions from mid-pregnancy to 11 years after delivery. There is no standard cut-point used to identify clinically relevant anxiety. The proportions of fathers with scores over 5 and 7 are shown in Table 3.1cb.

**Table 3.1cb.** Continuous measures of the CCEI to assess current anxiety, giving the proportions with scores over 5, and over 7.

Time	Variable	N	% >5	% >7
18wk gestation	PB233	9673	16.6	7.6
2 months	PC083	8205	12.0	5.9
8 months	PD174	7086	9.4	4.6
21 months	PE263	6034	11.9	5.7
33 months	PF4100	5297	16.1	7.2
6 years	PJ2100	4413	22.8	11.2
11 years	PP4050	3590	12.5	7.1

N = no. completed

*3.1d. Eating disorders*

Eating disorders can be serious conditions related to persistent eating behaviours that negatively impact health, emotions and ability to function. The most common of these are anorexia nervosa and bulimia nervosa. Men are far less prone to these disorders than women. The questions on anorexia and bulimia were asked on three occasions. Only bulimia had sufficient positive responses to present here ( Table 3.1d).

**Table 3.1d.** History of whether father had ever had bulimia.

Time asked	Variable name	N	Yes %	Never %
Pregnancy	PA172	7817	0.5	99.5
8 years	PL1042	3961	0.5	99.5
11 years	PP2002	3615	0.4	99.6

N = no. completed

*3.1e. Other psychiatric problems*

Fathers were asked whether they had any other psychiatric problems on 3 occasions ( Table 3.1e). Fewer than 3% reported such problems. They were described as text which can be accessed and coded directly by any interested researcher.

**Table 3.1e.** Prevalence (%) of other psychiatric problems in study fathers distinguishing between recently (in past year) and prior to the past year only.

Time asked	Variable name	N	Yes		No
			Recently %	In past %	Never %
Pregnancy	PA192	8435	0.2	1.4	98.4
8 years	PL1062	3940	1.0	1.8	97.2
11 years	PP2022	3581	1.0	1.7	97.3

N = no. completed

***3.2. Neurological disorders***

*3.2a. Headache / migraine*

Headache is pain in any region of the head. Headaches may occur on one or both sides of the head, be isolated to a certain location, radiate across the head from one point, or have a vicelike quality. A migraine is a headache that can cause severe throbbing pain or a pulsing sensation, usually on one side of the head. It's often accompanied by nausea, vomiting, and extreme sensitivity to light and sound.

Because there is often confusion concerning the distinction between headache and migraine, the ALSPAC fathers were usually asked about the occurrence of ‘headache or migraine’, although they were also asked about migraine itself. This showed a prevalence of 29–30% ( Table 3.2aa) and little sense of any trend with time. For headaches and/or migraine, over half the fathers were reported to have been affected within the various periods of time, and this was consistent between the report of the father himself and of the mother’s report ( Table 3.2ab). The frequency with which the father had headaches during a one-month period ( Table 3.2ac) shows that no more than 1% reported continual headaches, but that this rate did show a sign of increasing with time from 0.5% at 21 months to 1.0% at 12 years after the birth of the study child.

**Table 3.2aa.** History of whether father had ever had migraine distinguishing between recently (in past year) and prior to the past year only.

Time asked	Variable name	N	Yes		No
			Recently %	In past %	Never %
Pregnancy	PA177	8402	9.2	19.3	71.6
8 years	PL1047	3969	13.2	16.8	70.0
11 years	PP2007	3616	10.8	17.9	71.3

N = no. completed

**Table 3.2ab.** Prevalence (%) of headache or migraine in study fathers and if so whether they consulted a doctor.

Time asked	Variable name	N	Period covered	And consulted Dr.		Not at all
				Yes %	No %	%
*Paternal report*						
8m	PD022	6827	Since baby was born	2.3	43.9	53.9
21m	PE022	6151	Since baby was 8m	2.6	49.8	47.7
4y	PG1012	4987	In past year	3.1	58.0	39.0
5y	PH1012	4437	In past year	2.7	53.0	44.3
6y	PJ3012	4434	Since child’s 5 ^th^ birthday	2.7	52.5	44.8
9y	PM1012	3626	Since child’s 6 ^th^ birthday	4.4	57.4	38.2
12y	PQ1012	3218	In the last 2 years	3.8	56.7	39.5
*Maternal report*						
8m	F515	10615	Since baby’s birth	2.6	43.1	54.3
21m	G601	9209	Since baby was 8m	2.9	47.4	49.7
33m	H486	9026	Since baby was 18m	3.8	53.5	42.7
4y	J604	8442	In past year	3.9	56.1	40.0
6y	L6020	8101	Since child was 5	3.3	62.9	33.8
9y	P3020	6876	In last 2 years	4.6	65.6	29.8
11y	S3020	6748	Since child’s 10 ^th^ birthday	3.6	59.3	37.1

N = no. completed

**Table 3.2ac.** Frequency (%) of headache or migraine in study fathers in the past month.

Time asked	Variable name	N	Always %	Sometimes %	Not at all %
21m *	PE111	6140	0.5	56.2	43.3
4y	PG1131	4997	0.6	53.9	45.5
5y *	PH1151	4303	0.5	58.4	41.1
6y	PJ3241	4305	0.7	56.0	43.3
9y	PM1191	3639	0.8	52.5	46.7
11y	PP2141	3618	0.9	46.2	52.8
12y	PQ1191	3255	1.0	52.4	46.6

N = no. completed;

*Question included an option ‘once only’. This has been combined with ‘sometimes’

*3.2b. Epilepsy*

Epilepsy is a central nervous system (neurological) disorder in which brain activity becomes abnormal, causing seizures or periods of unusual behaviour, sensations and sometimes loss of awareness. At the time of the child’s birth 1% reported a history of epilepsy, although fewer (0.2%) reported having it recently ( Table 3.2b).

**Table 3.2b.** History of whether father had ever had epilepsy distinguishing between recently (in past year) and prior to the past year only.

Time Asked	Variable name	N	Yes		No
			Recently %	In past %	Never %
Pregnancy	PA175	8490	0.2	0.8	99.0
8 years	PL1045	3969	0.4	0.8	98.8
11 years	PP2005	3623	0.3	0.6	99.1

N = no. completed

*3.2c. Fatigue*

Although most people are over-tired occasionally, unrelenting exhaustion, on the other hand, lasts longer, is more profound and cannot be relieved by rest. Study fathers were asked on 7 occasions how often they had felt exhausted in the past month. In general, this was reported to have occurred almost all the time by 3–4% of fathers ( Table 3.2ca).

**Table 3.2ca.** Frequency (%) of fatigue in study fathers in the past month.

Time asked	Variable name	N	Always %	Sometimes %	Not at all %
21m *	PE119	6140	3.5	59.5	37.0
4y	PG1139	4995	3.6	57.0	39.4
5y *	PH1159	4489	3.6	64.5	31.9
6y	PJ3249	4442	5.1	61.6	33.3
9y	PM1199	3650	3.7	62.1	34.2
11y	PP2149	3615	3.3	50.7	45.9
12y	PQ1199	3262	3.1	49.2	47.8

N = no. completed; *Question included an option ‘once only’. This has been combined with ‘sometimes’

There was also a question on two occasions concerning a history of chronic fatigue syndrome (also known as CFS/ME) a disorder characterised by extreme fatigue which cannot be fully explained by an underlying medical condition. This was reported by 1.1 – 1.3%, with 0.3–0.4% stating that it had been present within the past year ( Table 3.2cb).

**Table 3.2cb.** History of whether father had ever had chronic fatigue syndrome distinguishing between recently (in past year) and prior to the past year only.

Time asked	Variable	N	Yes		No
			Recently %	In past %	Never %
8 years	PL1046	3960	0.4	0.9	98.7
11 years	PP2006	3613	0.3	0.8	98.9

N = no. completed

***4. Other health conditions***

A large number of mothers and the fathers reported other conditions that had occurred to the fathers over time ( Table 4). These conditions have been stored verbatim as text responses and can be accessed for coding by *bona fide* researchers.

**Table 4.** Prevalence (%) of other conditions in study fathers and if so whether they consulted a doctor.

Time asked	Variable name		Period covered	And consulted Dr.		Not at all
				Yes %	No %	%
*Paternal report*						
8m	PD036	6830	Since baby was born	6.1	2.5	91.4
21m	PE036	6148	Since baby was 8m	7.0	3.1	90.0
4y	PG1026	4573	In past year	13.8	3.5	82.7
5y	PH1036	4237	In past year	9.9	1.9	88.2
6y	PJ3037	4462	Since child’s 5 ^th^ birthday	10.0	2.2	87.8
9y	PM1037	3665	Since child’s 6 ^th^ birthday	10.5	1.7	87.8
12y	PQ1037	3297	In the last 2 years	11.0	2.9	86.1
*Maternal report*						
8m	F534	10615	Since baby’s birth	6.0	2.2	91.8
21m	G622	7107	Since baby was 8m	11.7	2.6	85.7
33m	H507	9026	Since baby was 18m	10.3	1.9	87.8
4y	J625	6807	In past year	16.6	2.1	81.3
6y	L6043	7062	Since child was 5	8.6	1.6	89.8
9y	P3043	7509	In past 2 years	8.9	1.3	89.8
11y	S3043	6767	Since child’s 10 ^th^ birthday	9.3	1.6	89.1

N = no. completed

## Strengths and limitations of the data on paternal measures of health

The participants recruited to the study were broadly representative of the general population of parents resident in the area at the time in terms of sex, ethnicity and socio-economic status ( Fraser *et al.*, 2013). The data described here can be linked to all the other data collected throughout the study. This includes information about the relationships between partners and the study child, biological markers from different members of the family, data regarding the parents’ beliefs and behaviours, physical and psychological environments, life experiences and demographics. Information was also collected from study fathers before and during the pandemic and is currently ongoing. The data can also be linked to data collected on fathers who were clinically examined in 2011–13 and will be able to be linked to details of ongoing clinical examinations ( https://ahrp.blogs.bristol.ac.uk/).

In regard to the data collected on mental health, the scales used to measure depression and anxiety have been extensively used and validated. Nevertheless, as Reviewer 1 has pointed out, the EPDS has been criticized as not sufficient to modify the cut-off scores in fathers (as much as necessary) ( Matthey, 2022). It is limited to evaluating depressive and anxious symptoms, without considering that fathers, and males in general, tend to manifest affective symptoms in a different way from mothers, for example with behavioural acting out and externalizing disorders (anger attacks, violent or dangerous conduct, compulsive physical or sexual activity, extramarital affairs, running away from home or at work, relationship conflicts). Although there are now instruments that are better able to capture such manifestations of male depression, they were not available at the time that ALSPAC was designed. The benefit of the EPDS as used here is that the same instrument is measured over time.

Similarly, identification of physical disorders, although somewhat crude, have the advantage of being recorded over time using identical wording and, in addition, have the comparison of maternal report of her partner’s disorders to compare with his own reports.

A limitation of this study is the lack of diversity, because at the time of enrolment, the population of Avon was mainly Caucasian, therefore there were too few Black, Asian and Minority Ethnic (BAME) participants (<6%) to allow for detailed analysis by ethnic background. In addition, as with all longitudinal studies, there is a loss to follow up over time, either through participants moving and failing to notify the study, dying or, rarely, withdrawing their consent for the study. In the latter case the data are removed.

In this paper we have described changes over time. However, the reader should be aware that the changes could be the result of differential response to the completion of the relevant questionnaires or due to a change of the mother’s partner; however, these can be removed from relevant questionnaire time points using an appropriate variable in the PZ file (partner_changed_when). It should also be emphasised that the reports are from the fathers and mothers. Whereas their reports are likely to be fairly accurate in regard to signs and symptoms, diagnoses are probably less accurate as they have not been validated by linkage to clinical records.

The influence of the father has been much neglected in considering the dynamics of the family over time, in contrast to the considerable amount of research that has considered the mother’s health – particularly her mental health. By describing the measures of physical and mental health that are available we hope to encourage research into the ways in which aspects of the fathers’ health contributes to the health and development of children and the well-being of mothers. We also are aware that these data will be important in the long-term follow-up of these fathers over time.

ALSPAC publications on fathers’ health have concentrated on paternal depression and anxiety to date (e.g. Ben-Shlomo *et al.*, 2016; Capron *et al.*, 2015; Gutierrez-Galve *et al.*, 2015; Gutierrez-Galve *et al.*, 2019; Hanington *et al.*, 2010; Hanington *et al.*, 2012; Heron *et al.*, 2004; Hibbeln *et al.*, 2018; Nawa *et al.*, 2021; Ramchandani *et al.*, 2005; Ramchandani *et al.*, 2006; Ramchandani *et al.*, 2008a; Ramchandani *et al.*, 2008b; van Batenburg-Eddes *et al.*, 2013) but data are available for other features of paternal health.

## Ethical approval and consent

Prior to commencement of the study, approval was sought from the ALSPAC Ethics and Law Committee and the Local Research Ethics Committees. Informed consent for the use of data collected via questionnaires and clinics from participants was assumed if they had voluntarily completed a questionnaire following the recommendations of the ALSPAC Ethics and Law Committee at the time. Questionnaires were completed in the participants’ own home and return of the questionnaires was taken as continued consent for their data to be included in the study ( Birmingham, 2018). Full details of the approvals obtained are available from the study website ( http://www.bristol.ac.uk/alspac/researchers/research-ethics/). Study members have the right to withdraw their consent for elements of the study or from the study entirely at any time.

## Data Availability

ALSPAC data access is through a system of managed open access. The steps below highlight how to apply for access to the data included in this paper and all other ALSPAC data. Please read the ALSPAC access policy ( http://www.bristol.ac.uk/media-library/sites/alspac/documents/researchers/data-access/ALSPAC_Access_Policy.pdf) which describes the process of accessing the data and biological samples in detail, and outlines the costs associated with doing so. You may also find it useful to browse our fully searchable research proposals database ( https://proposals.epi.bristol.ac.uk/), which lists all research projects that have been approved since April 2011. Please submit your research proposal ( https://proposals.epi.bristol.ac.uk/) for consideration by the ALSPAC Executive Committee using the online process. You will receive a response within 10 working days to advise you whether your proposal has been approved. If you have any questions about accessing data, please email: alspac-data@bristol.ac.uk (data) or bbl-info@bristol.ac.uk (samples).
