# Supplementary material for: Paternal health in the first 12-13 years of the ALSPAC study
Source: Wellcome Open Res. 2024 Oct 11;8:8. Originally published 2023 Jan 6. [Version 3] doi: 10.12688/wellcomeopenres.18639.3 (PMC10354460; doi:10.12688/wellcomeopenres.18639.3)
Supplement: Peer review history

# Reviewer report: Reviewer response for version 3

**DOI:** 10.21956/wellcomeopenres.25571.r106761

**Published:** 2024-11-26

**Authors:** Rachel C. Whooten

**Affiliations:** 1 Massachusetts General Hospital for Children, Boston, Massachusetts, USA

Provides descriptive characteristics of fathers from ALSPAC study related to variety of different physical and mental health conditions. I think that this data is valuable in guiding future researchers. Agree with recommendation of prior reviewer relating to potential uses for this information. This information from fathers will be helpful in moving the literature forward.

Are sufficient details of methods and materials provided to allow replication by others?

Yes

Is the rationale for creating the dataset(s) clearly described?

Yes

Are the datasets clearly presented in a useable and accessible format?

Yes

Are the protocols appropriate and is the work technically sound?

Yes

Reviewer Expertise:

Early childhood health behavior interventions

I confirm that I have read this submission and believe that I have an appropriate level of expertise to confirm that it is of an acceptable scientific standard.

# Reviewer report: Reviewer response for version 3

**DOI:** 10.21956/wellcomeopenres.25571.r108314

**Published:** 2024-11-22

**Authors:** Rhonda C. Bell, Patrick Lightning Jr.

**Affiliations:** 1 Division of Human Nutrition, University of Alberta, Alberta, Canada; 2 University of Alberta, Edmonton, AB, Canada

As noted by others, this article provided data on paternal health characteristics across time from the Avon Longitudinal Study of Parents and Children (ALSPAC) Study.  The information presented provides a picture of how study fathers’ self-perceived health has changed over time. Generally the cohort of study fathers are relatively healthy. 

 There are interesting findings that may lead to additional important investigations.  For example, among those who reported experiencing physical or emotional challenges or illness, few sought advice from a doctor. Since participants did not seek medical advice, how did they addressed these challenges?

 In the strengths and limitations section, it would be helpful to recognize that the approach that was taken with these questions and questionnaires, is generally deficit-focused.   Future rounds of data collection could consider asking about ways that participants effectively dealt with depression, anxiety, etc.  This information would provide a more rounded view of how study fathers might mediate difficult times.

Are sufficient details of methods and materials provided to allow replication by others?

Yes

Is the rationale for creating the dataset(s) clearly described?

Yes

Are the datasets clearly presented in a useable and accessible format?

Yes

Are the protocols appropriate and is the work technically sound?

Yes

Reviewer Expertise:

Human nutrition, maternal health in preconception, pregnancy and postpartum, supporting family health

We confirm that we have read this submission and believe that we have an appropriate level of expertise to confirm that it is of an acceptable scientific standard.

# Reviewer report: Reviewer response for version 3

**DOI:** 10.21956/wellcomeopenres.25571.r104596

**Published:** 2024-11-13

**Authors:** Franco Baldoni

**Affiliations:** 1 University of Bologna, Bologna, Italy

The article has now been improved and can be indexed in its current form (Version 3)

Are sufficient details of methods and materials provided to allow replication by others?

Partly

Is the rationale for creating the dataset(s) clearly described?

Yes

Are the datasets clearly presented in a useable and accessible format?

Yes

Are the protocols appropriate and is the work technically sound?

Yes

Reviewer Expertise:

Father, Perinatal mental health, Attachment

I confirm that I have read this submission and believe that I have an appropriate level of expertise to confirm that it is of an acceptable scientific standard.

# Reviewer report: Reviewer response for version 1

**DOI:** 10.21956/wellcomeopenres.20668.r58196

**Published:** 2023-07-18

**Authors:** Linda M. Richter

**Affiliations:** 1 DSI-NRF Centre of Excellence in Child Development, University of the Witwatersrand, Johannesburg, South Africa

The data brief provides percentages of fathers in ALSPAC who were reported by themselves, or their partners (the mothers) to have physical problems, as determined subjectively, eight times between pregnancy (up to 8 months of child age) and child age 12 years.

 Physical problems cover a wide range of symptoms, illnesses and ailments from arthritis to epilepsy, depression and fatigue. Frequencies are reported for mother and father responses combined and for all time periods combined. Thus some of the conclusions drawn are not justified by the data available to the reader – for example, whether mothers report physical problems more often than men do, or that reports of physical problems increase with age.

 The data brief would be more valuable if, for each ailment, frequencies were given for reports by mothers and fathers separately, and by age. These additions would also show variations in responses over time.

Are sufficient details of methods and materials provided to allow replication by others?

Partly

Is the rationale for creating the dataset(s) clearly described?

Yes

Are the datasets clearly presented in a useable and accessible format?

Yes

Are the protocols appropriate and is the work technically sound?

Partly

Reviewer Expertise:

Child and adolescent development, birth cohort studies

I confirm that I have read this submission and believe that I have an appropriate level of expertise to confirm that it is of an acceptable scientific standard, however I have significant reservations, as outlined above.

# Response

**Published:** 2024-02-09

**Authors:** Jean Golding

**Affiliations:** Bristol Medical School, University of Bristol, Bristol, UK

Thank you very much for your suggestion to indicate ways in which these data may be useful to various researchers using the various ALSPAC data sets. We have therefore added this paragraph to the end of the paper:  The influence of the father has been much neglected in considering the dynamics of the family over time, in contrast to the considerable amount of research that has considered the mother’s health – particularly her mental health. By describing the measures of physical and mental health that are available we hope to encourage research into the ways in which aspects of the fathers’ health contributes to the health and development of children and the well-being of mothers. We also are aware that these data will be important in the long-term follow-up of these fathers over time.

# Reviewer report: Reviewer response for version 1

**DOI:** 10.21956/wellcomeopenres.20668.r59742

**Published:** 2023-07-14

**Authors:** Sharin Baldwin

**Affiliations:** 1 London North West University Healthcare Trust, London, UK; 2 University of Warwick, Warwick, UK

This article provides data from the Avon Longitudinal Study of Parents and Children (ALSPAC) study on paternal physical and mental health from pregnancy to 12 years after birth. The data presented are mainly descriptive which can be useful for researchers to link to other studies. It would be helpful to have more detail as to why this article has been written and how useful this information could be to inform or support other studies. Examples could be given for the different ways in which this dataset could be utilised. Although the authors have provided a brief explanation, I think this could be expanded further to make the importance of this dataset more explicit.

 It would also be good to add some demographic details (i.e. age, education/occupation etc) of the fathers in the study to provide the readers with a better understanding of the sample. 

 Overall, I think this data note is an important addition to the information available from the ALSPAC study.

Are sufficient details of methods and materials provided to allow replication by others?

Yes

Is the rationale for creating the dataset(s) clearly described?

Partly

Are the datasets clearly presented in a useable and accessible format?

Yes

Are the protocols appropriate and is the work technically sound?

Yes

Reviewer Expertise:

Men' transition to fatherhood, Paternal mental health, Health visiting/ child health

I confirm that I have read this submission and believe that I have an appropriate level of expertise to confirm that it is of an acceptable scientific standard.

# Reviewer report: Reviewer response for version 1

**DOI:** 10.21956/wellcomeopenres.20668.r59746

**Published:** 2023-07-14

**Authors:** Franco Baldoni

**Affiliations:** 1 University of Bologna, Bologna, Italy

The article, in accordance with the editorial lines of Wellcome Open Research, deals with updated data from the Avon Longitudinal Study of Parents and Children (ALSPAC), an important longitudinal study carried out in Avon, UK, on a population of over 14,000 mothers together to their partners (even non-biological fathers) and children. The study, which began in 1991-1992, has been previously documented by numerous articles. The goal was to assess how children's health, well-being and development was affected by the environment in which they grew up. A key feature of the ALSPAC is that it is one of the few longitudinal studies that also assesses the characteristics of the mother's partner (regardless of their gender or parental status). The study was carried out by administering self-report questionnaires from pregnancy up to the age of 12-13 of the children.

 This article mainly illustrates the data relating to the physical and mental health of the mothers' partners (defined as study fathers).

 The aim of the study is well introduced, the methodology is clearly described, satisfactorily defining the symptoms and pathologies studied. The description of the data is satisfactorily illustrated by numerous tables, even if not much space has been reserved for discussion.

 Despite this, the authors are advised to integrate the text with some clarifications, to be possibly included within the limits of the study.

1. In the part dedicated to Psychiatric and Neurological conditions (pp. 23-27) it is specified that maternal and paternal depression in ALSPAC were assessed with the use of the EPDS (p. 25). Although this instrument has also been considered valid for screening for paternal perinatal affective disorders, it has also been criticized as not sufficient to modify the cut-off scores in fathers (as much as necessary) (Matthey, 2022). The EPDS is limited to evaluating depressive and anxious symptoms, without considering that fathers, and males in general, tend to manifest affective symptoms in a different way from mothers, for example with behavioral acting out and externalizing disorders (anger attacks, violent or dangerous conduct, compulsive physical or sexual activity, extramarital affairs, running away from home or at work, relationship conflicts). This requires the adoption of a gender-based perspective (Baldoni and Giannotti, 2020) using, if possible, validated tools developed for this purpose such as the *Ddads Questionnaire *(Vermeulen and Buyl, 2021) and the *Perinatal Assessment of Paternal Affectivity *(PAPA) (Baldoni et al., 2022). Obviously when the ALSPAC was designed and started (1991-1992) this problem was not yet evident and sufficiently studied, but it could be at least mentioned and discussed within the limits of the study.
2. We appreciated that the study evaluated the possible addictions of fathers, an index associated with paternal affective disorders). Another useful data regarding Psychiatric and Neurological conditions, which can be added, if available, is whether the study fathers took psychotropic drugs and when.

In conclusion, the article is of high scientific interest, well written, compliant with the scientific literature and could be published later after making the suggested additions.

 References:

1. Baldoni, F. & Giannotti, M. (2020). Perinatal distress in fathers: toward a gender-based screening of paternal perinatal depressive and affective disorders. *Frontiers in Psychology*, 11, 1-5. doi.org/10.3389/fpsyg.2020.01892
2. Baldoni, F., Giannotti, M., Casu, G., Agostini, F., Mandolesi, R., Peverieri, S., Ambrogetti, N., Spelzini, F., Caretti, V., & Terrone, G. (2022). The Perinatal Assessment of Paternal Affectivity (PAPA):  Italian validation of a new tool for the screening of perinatal depression and affective disorders in fathers. *Journal of Affective Disorders*, 317, 123-130. https://doi.org/10.1016/j.jad.2022.08.049
3. Matthey, S. (2022). Is Validating the Cutoff Score on Perinatal Mental Health Mood Screening Instruments, For Women and Men from Different Cultures or Languages, Really Necessary? Int. J. Environ. Res. *Public Health*, 19, 4011. https://doi.org/10.3390/ijerph19074011
4. Vermeulen, J, Buyl, R (2021). The development of the DDads questionnaire: Awareness, knowledge and attitudes of the general population towards paternal depression. *Front. Psychiatry* 11, DOI:10.3389/fpsyt.2020.561954

Are sufficient details of methods and materials provided to allow replication by others?

Partly

Is the rationale for creating the dataset(s) clearly described?

Yes

Are the datasets clearly presented in a useable and accessible format?

Yes

Are the protocols appropriate and is the work technically sound?

Yes

Reviewer Expertise:

Attachment, Psychotherapy, Psychosomatics, Paternal perinatal  affective disorders

I confirm that I have read this submission and believe that I have an appropriate level of expertise to confirm that it is of an acceptable scientific standard, however I have significant reservations, as outlined above.

## References

[1] : Perinatal Distress in Fathers: Toward a Gender-Based Screening of Paternal Perinatal Depressive and Affective Disorders. *Front Psychol* .2020;11: 10.3389/fpsyg.2020.01892 1892 10.3389/fpsyg.2020.01892 32973604 PMC7461929

[2] : The Perinatal Assessment of Paternal Affectivity (PAPA): Italian validation of a new tool for the screening of perinatal depression and affective disorders in fathers. *J Affect Disord* .2022;317: 10.1016/j.jad.2022.08.049 123-130 10.1016/j.jad.2022.08.049 36028014

[3] : Is Validating the Cutoff Score on Perinatal Mental Health Mood Screening Instruments, for Women and Men from Different Cultures or Languages, Really Necessary?. *Int J Environ Res Public Health* .2022;19(7) : 10.3390/ijerph19074011 10.3390/ijerph19074011 35409694 PMC8998276

[4] : The Development of the DDads Questionnaire: Awareness, Knowledge and Attitudes of the General Population Towards Paternal Depression. *Front Psychiatry* .2020;11: 10.3389/fpsyt.2020.561954 561954 10.3389/fpsyt.2020.561954 33551859 PMC7859093

# Response

**Published:** 2024-10-02

**Authors:** Jean Golding

**Affiliations:** Bristol Medical School, University of Bristol, Bristol, UK

Many thanks for this review of the problems with using the EPDS for measuring depression in men. We have now inserted your suggested text in the Limitations of the revised version of the Data Note. You comment on the lack of a detailed discussion. This is because of the publisher’s requirements for Data Notes. The instructions state: ‘Data Notes are brief descriptions of scientific datasets that promote the potential reuse of research data and include details of why and how the data were created; they do not include any analyses or conclusions’. Nevertheless we have added a paragraph at the end of the Discussion to highlight the possible uses of these data.
